# Mutations in the signal peptide of effector gene *Pi04314* contribute to the adaptive evolution of the *Phytophthora infestans*

**DOI:** 10.1186/s12862-025-02360-4

**Published:** 2025-03-14

**Authors:** Hai-Bing Ouyang, Yan-Ping Wang, Meng-Han He, E-Jiao Wu, Bin-Hong Hu, Jiasui Zhan, Lina Yang

**Affiliations:** 1 Fujian Key Laboratory on Conservation and Sustainable Utilization of Marine Bioaffiliationersity, Fuzhou Institute of Oceanography, Minjiang University, Fuzhou, 350108 China; 2 Department of Plant Pathology, Nanjing Agricultural University, Nanjing, 210095 Jiangsu China; 3 Sichuan Provincial Key Laboratory for Development and Utilization of Characteristic Horticultural, Biological Resources, College of Chemistry and Life Sciences, Chengdu Normal University, Chengdu, China; 4 College of Plant Protection, Henan Agricultural University, Zhengzhou, 450002 China; 5 Institute of Pomology, Jiangsu Key Laboratory for Horticultural Crop Genetic Improvement, Jiangsu Academy of Agricultural Sciences, Nanjing, 210014 China; 6 Department of Forest Mycology and Plant Pathology, Swedish University of Agricultural Sciences, Uppsala, Sweden

**Keywords:** Pathogen evolution, Effector gene, Mutation mechanism, Population genetics, Disease epidemiology

## Abstract

**Background:**

Effectors are critical in the antagonistic interactions between plants and pathogens. However, knowledge of mutation mechanisms and evolutionary processes of effectors remains fragmented despite its importance for the sustainable management of plant diseases. Here, we used a population genetic approach to explore the evolution of the effector gene *Pi04314* in *Phytophthora infestans*, the causal agent of potato blight.

**Results:**

We found that *Pi04314* gene exhibits a low genetic variation generated by point mutations mainly occurring in the signal peptide. Two of the 14 amino acid isoforms completely abolished the secretion functions of signal peptides. The effector is under purifying selection, supported by the comparative analyses between its population differentiation with that of SSR marker loci as well as by negative Tajima’s D (-1.578, *p* = 0.040) and Fu’s FS (-10.485, *p* = 0.000). Furthermore, we found that the nucleotide diversity of *Pi04314* is significantly correlated with the annual mean temperature at the collection sites.

**Conclusion:**

These results suggest that the evolution of effector genes could be influenced by local air temperature and signal peptides may contribute to the ecological adaptation of pathogens. The implications of these results for agricultural and natural sustainability are discussed.

## Background

Pathogen and host interactions are shaped by the constant genomic changes in both partners [1, 2]. Such interactions can lead to either pathogenic or non-pathogenic outcomes but their trajectory can be altered by mutation and natural selection that change the status and prevalence of genes associated with host susceptibility and pathogen pathogenicity [3, 4]. Beneficial mutations that can escape host immune systems or recognize pathogen infection can be gradually accumulated in pathogen or host populations through adaptive selection while harmful mutations that have detrimental effects on host immunity or pathogen pathogenicity will be eliminated by negative selection. On the other hand, the prevalence and richness of neutral mutations which do not exert effect on host immunity or pathogen pathogenicity are mainly maintained in populations by drift-migration dynamics [5].

In the plant kingdom, many host-pathogen interactions follow the gene-for-gene model in which effectors, a group of small proteins secreted by pathogens [6–8], have crucial impacts on the antagonistic coevolution between pathogens and plants by shaping the direction and landscape of interactions [2, 9]. Effectors usually act as ligands to regulate enzyme activity, gene expression and/or cell signaling of the plant host, to establish a favorable environment for disease establishment or directly modify the activity of some plant mRNA molecules [10, 11]. They are usually subjected to epigenetic regulation mediated by repetitive elements, sRNA [12] and environmental factors [13]. At the first confrontation between pathogen and host, conserved pathogen-associated molecular patterns (PAMPs/MAMPs) are recognized by cell-surface pattern recognition receptors (PRRs) in plants to activate pattern-triggered immunity (PTI), which restricts pathogenicity [14]. On the other hand, host-adapted pathogens have developed antagonistic mechanisms by secreting effectors to either host extracellular space or living cells to suppress PTI. In the next phase of the interaction, effectors are recognized by resistance (R) proteins in the host plants to elicit a rapid effector-triggered immunity response (ETI), which often is accompanied by a cell death and then restrict pathogen infection [15, 16]. Pathogens then evolve to diversify or even lose effectors to suppress or evade ETI.

Pathogens generally have a shorter generation time and larger population size than their plant hosts [17, 18]. Furthermore, many filamentous pathogens have a mixed model of sexual and asexual reproduction [19]. These demographic and biological features place pathogens in adaptive advantage over their plant hosts in antagonistic interaction. In particular, modern agriculture is characterized by monoculture, multiple-cropping and international trading of production and plant materials [20]. Many plant pathogens have evolved at an accelerated rate in agricultural systems due to the strong selective pressures imposed by the large-scale uninterrupted cultivation of uniform plant genotypes and increasing exchange of genetic material and novel pathogenicity traits associated with globalization [21, 22]. Escalating pathogen evolution may contribute to the rapid loss of disease management approaches including the efficacy of vertical resistance mediated by gene for gene interaction [23].

*Phytophthora infestans*, the causal agent of potato late blight, is known as a plant killer that once led to the Irish potato famine in the 19th century [24, 25]. The annual global economic losses caused by *P. infestans* are estimated to be $5 billions [26], posing a great threat to food security and social economics. The pathogen can successfully colonize plant hosts by delivering effector proteins that suppress potato defenses and increase disease severity [10, 27–30]. At least 563 RXLR (Arg-x-Leu-Arg) effectors, named due to the conserved Arginine-any amino acid-Leucine-Arginine motif that follows the signal peptide of the proteins, have been predicted in the *P. infestans* genome. The gene-sparse and repeat-rich regions facilitate rapid adaptive evolution of effector genes, enabling quick overcoming of the corresponding resistance genes [31–33]. Molecular cloning and functional characterization of *P. infestans* effectors show that signal peptides direct effector protein secretion and transport to plant cells by way of Golgi apparatus and endoplasmic reticulum [34]. Signal peptides are usually cleaved off after entering into plant cells and then presumably rapidly degraded, but some of them may remain and serve other functions [35, 36]. However, the mutation mechanisms and evolutionary processes governing signal peptide in effector genes is poorly understood.

According to their interaction with potato plants, effector genes can be divided into three categories including: (1) long-term challenged by corresponding resistance genes from *Solanum tuberosum* that have been introgressed into potato varieties and widely deployed in the field such as *Avr1-Avr11* [37–39]; (2) corresponding resistance genes already known but have not been incorporated into potato cultivars or have not been widely used in the field such as the *Avripio* family, *Pi22825*,* Pi02860 and Pi04373* [40, 41]; and (3) being cloned recently or annotated but their corresponding resistance genes have not yet been identified such as *Pi04314*, *Pi04089*, *Pi_22798*, *Pi15718.2*, and *Pi03192* [28, 42–46].

*Pi04314* is a classical RXLR effector of *P. infestans*, which is strongly upregulated during the biotrophic phase of infection [28]. It is predicted that *Pi04314* contains an N-terminal signal peptide, a conserved RxLR domains, and a WY structural fold characteristic [47]. Functional analysis of pathogenicity shows that Pi04314 interacting with susceptibility factors forms a holoenzyme complex to promote late blight disease development. The complex enhances leaf colonization of *P. infestans* via activity in the host nucleus and attenuates induction of jasmonic and salicylic acid-responsive genes [28].

Research in effector genes of plant pathogens including *P. infestans* mainly focuses on their functional characterizations of pathogenicity. Population genetic analyses of effector genes in large pathogen samples from diverse ecological backgrounds are limited but important for understanding many unanswered questions related to antagonistic coevolution and have practical implications for disease management and food security. These issues are of great public concern in the era of ongoing climate change and burgeoning global population [48, 49]. Therefore, the specific objectives of this study were to: (1) analyze the genetic diversity and spatial distribution of *Pi04314* in *P. infestans* population, (2) understand the evolutionary mechanisms defining the population genetic structure of *Pi04314*, and (3) evaluate the role of signal peptide and air temperature in the ecological adaptation of *P. infestans Pi04314*. To achieve these objectives, we used population genetic and phylogenetic approach to analyze the sequences character of *Pi04314* sampled from seven potato fields across China.

## Materials and methods

### Pathogen isolates and SSR genotyping

**Fig. 1:**
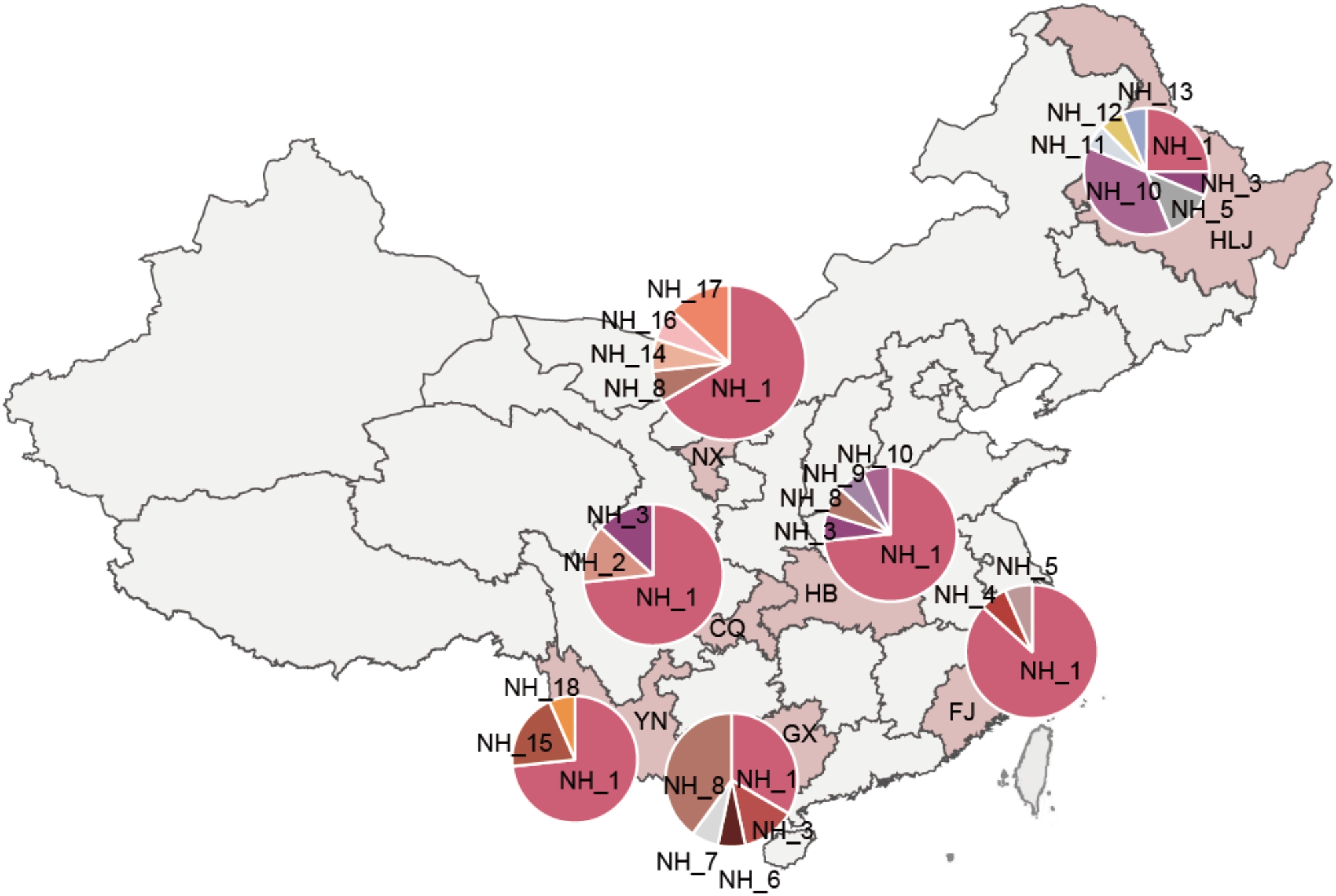
Frequency and spatial distribution of *Pi04314* nucleotide haplotypes in the *Phytophthora infestans* populations sampled from seven geographic locations of potato fields in China

*Phytophthora infestans* isolates were collected from potatoes across seven regions in China, including Yunnan, Guangxi, Fujian, Chongqing, Hubei, Ningxia, and Heilongjiang, during the middle stage of epidemics from 2010 to 2013 (Fig. 1). Briefly, infected leaves were randomly selected from plants spaced 1–2 m apart and transported to the laboratory for isolation within 24 h. The leaves were first washed with tap water for 60 s, followed by sterilized distilled water for 30 s and placed on 1% water agar with abaxial side up for 24 h. A single piece of hyphae was aseptically removed from the sporulating lesions with an inoculating needle, transfered to a rye B agar plate supplemented with rifampicin (10 µg/ml) and ampicillin (100 µg/ml) and cultured at 19℃ in the dark for seven days to develop colonies. The isolates were purified by transferring single sporangium from the resulting colonies to a fresh rye B plate. Genomic DNA from each of the isolates was extracted and amplified with eight pairs of SSR primers (G11, Pi02, Pi04, Pi4B, Pi16, Pi33, Pi56, and Pi89) as described previously [50–52]. The PCR products were labeled with fluorescent dyes in a thermal cycler (Applied Biosystems, USA) and sent to Ruiboxingke Biotechnology Company Limited (Beijing, China) for fragment analysis with an ABI 3730XL automated DNA sequencer (Applied Biosystems, Foster, California). Alleles were assigned using GeneMarker software version 3.7 with a binning procedure, and multi-locus genotypes were formed by joining alleles at each SSR locus in the same order. The isolates were further genotyped by restriction enzyme-PCR amplification of mitochondrial haplotypes, mating type [53, 54], and partial sequence analysis of three genes (b-tubulin, Cox1, and Avr3a) [55]. The details of pathogen isolation and molecular characterization can be found in our previous publications [56].

### *Pi04314* sequencing

A total of 106 isolates were sequenced for the *Pi04314* gene. To produce fungal tissue for DNA extraction, isolates were retrieved from long-term storage and cultured on rye B agars at 19℃ in the dark for 7 days. Mycelia from each isolate were harvested, transferred into a sterile, 2 ml centrifuge tube, and lyophilized by a vacuum freeze dryer (Alpha1-2, Christ, Germany). The lyophilized mycelia were ground to powder with a mixer mill (MM400, Retsch, Germany), and genomic DNA was extracted using a plant genomic DNA kit (Promega Biotech. Co. TRANSGEN., Beijing) according to the manufacturer’s instructions. Approximately 0.1 g mycelia were used for DNA extraction of each isolate. The resulting DNA was suspended in ultrapure water and kept in a -40℃ refrigerator until use.

Genomic DNA was amplified with a pair of *Pi04314* specific primers (F: 5’-CCATCTTAACAAGTCCAAAG-3’, R: 5’-CGTTCAGGCGTAGGTGTTTT-3’) designed based on the T30-4 sequences. PCR amplification was performed in a total volume of 25 µl reaction buffer composed of 2.5 µl of 10×Buffer, 2.0 µl of dNTPs (2.5 mM), 1.0 µl of forward primer (10 µmol/l), 1.0 µl of reverse primer (10 µmol/l), 17.3 µl of ddH_2_O, 0.2 µl of Taq polymerase (TaKaRa Ex Taq^®^), and 2.0 µl of template cDNA. The PCR program started with an initially denatured at 94℃ for 5 min; followed by 35 cycles of 94℃ for 30 s, 55℃ for 30 s and 72℃ for 50 s; and ended with an extension at 72℃ for 15 min. PCR products were separated by electrophoresis and purified. They were then ligated into a T5 zero cloning vector and transformed into competent cells *Trans*1-T1 by heat shock at 42℃ for 30 s according to the manufacturer’s instructions (pEASY^®^-T5 Zero Cloning Kit). Six colonies were picked from each transformation and incubated in LB liquid media with shaking overnight. Colonies with single and expected amplicon size were picked and sequenced using an ABI3730 automated DNA sequencer (Applied Biosystems, USA) by Thermo Fisher Scientific (Invitrogen, Shanghai, China).

### Population genetic analysis

Nucleotide sequences of *Pi04314* were aligned in MEGA5 [57] using the MUSCLE algorithm [58]. Haplotypes were reconstructed by the PHASE algorithm implemented in DnaSP v5 [59]. The frequency of haplotype from different regions was compared by a contingency χ^2^ test. Nucleotide diversity (Pi), haplotype diversity (Hd), and population differentiation were estimated by DnaSP v5. SSR data were extracted from previous publications [50, 51, 60] and population differentiation in the SSR marker loci was estimated using POPGENE 1.32 [61]. The average annual temperature at the collection sites was obtained from World Climate and presented as a mean value calculated over a period of 15 to 30 years.

### Haplotype networks construction

To reveal the genealogical relationships among haplotypes, a median-joining (MJ) network was generated using Network 5.0 [62]. This software identifies the shortest genetic genealogy by linking all haplotypes together with the least mutation steps.

### Detecting natural selection in *Pi04314*

Tajima’s D [63] and Fu’s FS [64] were used to evaluate the neutrality of the gene with Arlequin 3.5 [65]. These parameters compare the expected nucleotide diversity inferred from the number of segregating sites and the preserved nucleotide diversity in the sequences. If the two values are statistically different, the hypothesis of neutral evolution in the concerned genes is rejected. On the other hand, the concerned genes are considered to be exempted from natural selection. Positive Tajima’s D indicates an excess of alleles with middle frequency likely caused by balancing selection while negative Tajima’s D indicates an over-representation of alleles with low frequency caused by purifying selection. Natural selection in the *Pi04314* gene was also assessed by comparing its population differentiation with SSR markers using a t-test as described previously [66]. The standard deviation of population differentiation in the SSR markers was generated by bootstrapping 1000 resamples. A significant difference between the two population differentiations suggests that the *Pi04314* gene is under natural selection. Otherwise, population differentiation estimated from *Pi04314* and SSR markers should be similar.

PAML 4.7 [67] and HyPhy [68] were used to identify selection sites in the gene by calculating the ratio of nonsynonymous (dN) to synonymous (dS) substitution (ω = dN/dS). The detection involves six codon substitution models, i.e., M0 (one-ratio), M1a (nearly neutral), M2a (positive selection), M3 (discrete), M7 (beta), M8 (beta and ω > 1) and M8a (beta and ω = 1) and assumes that the ω ratio is the same across branches of the phylogeny but different among sites in the alignment. The models were evaluated by paired (i.e., M0 vs. M3, M1a vs. M2a, and M7 vs. M8 ) ratio tests (LRTs) using the CODEML algorithm and the model that has significant LRT (*p*-value < 0.01) and that best fits the data was determined [69]. For example, the site of positive selection can be inferred if M2a provides a better fit than M1a, or if M8 provides a better fit than M7 or M8a. In addition, SLAC and REL [70–72] implemented in HyPhy were also used to evaluate natural selection sites. The sites with a posterior probability of *p* < 0.05 and *p* > 0.95 for ω was considered to be under selection.

### Transient expression of proteins in yeast strain

To determine the function of signal peptide in *Pi04314*, Avr1bSP and signal peptide sequences (1–66 nucleotides) of the 18 *Pi04314* nucleotide haplotypes were inserted into plasmid pSuc2t7M13ori and transformed into yeast strain YTK12 [73], respectively. The transformants were screened on CMD/-W medium and YPRAA medium plates. The invertase activity of the transformants was evaluated by measuring their ability of reducing 2, 3, 5-triphenyltetrazolium chloride (TTC) to an insoluble, red-colored 1, 3, 5-triphenyl formazan (TPF). Transformants were cultured in liquid CMD/-W medium at OD600 of 0.4, and approximately 2 mL of cell suspension was collected and re-suspended with 250 µl of 10 mM acetic acid-sodium acetate buffer (pH 4.7), 500 µl of 10% sucrose solution (w/v) and 750 µl of sterile distilled water at 37℃ for 10 min. After centrifugation at 12,000 g for 1 min, 400 µl of the supernatant was transferred into a glass test tube containing 3.6 ml of 0.1% TTC solution at room temperature for 10 min.

## Results

### Sequence variation and spatial distribution in *Pi04314*

**Fig. 2:**
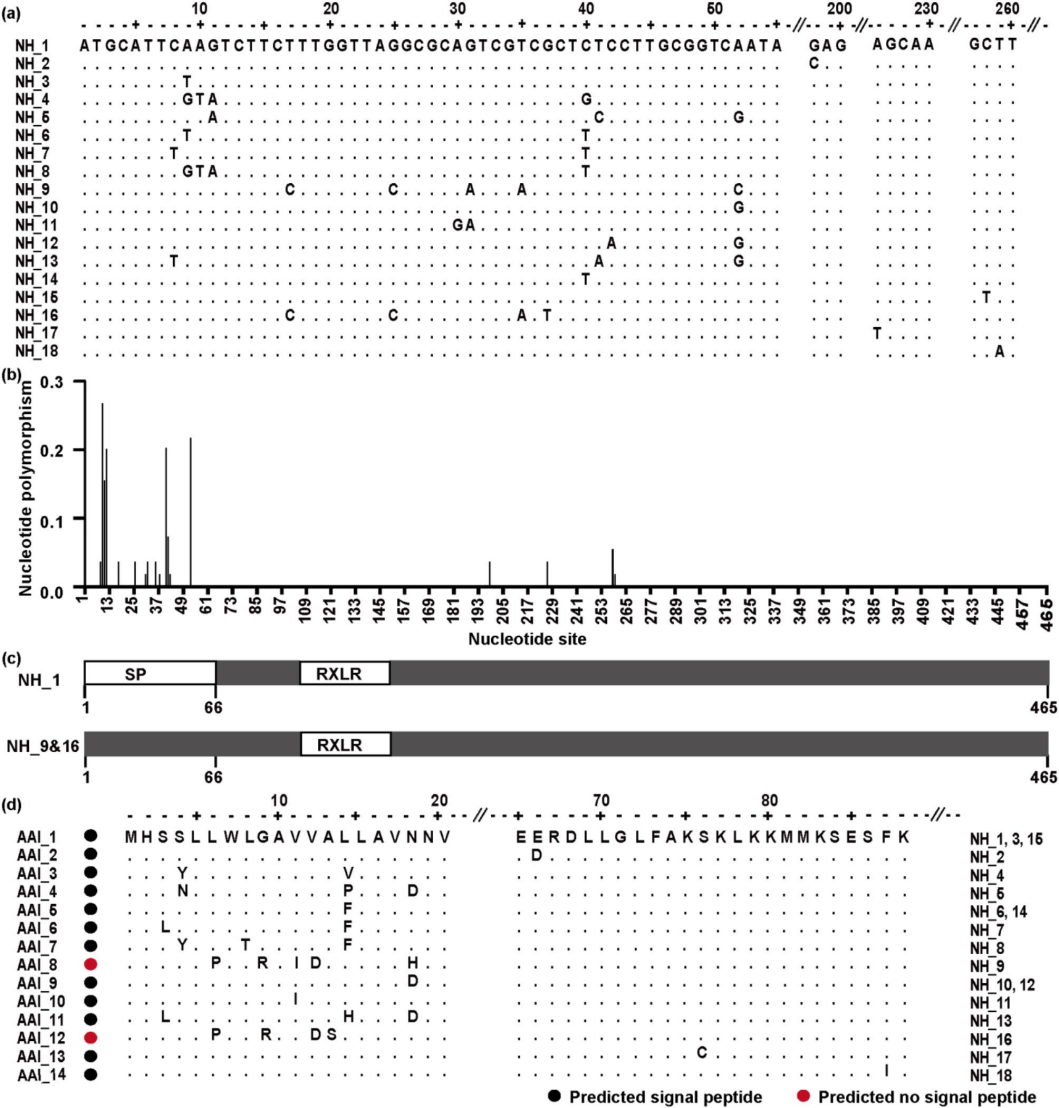
Mutation and distribution of *Pi04314*. (**a**) Sequence alignment of 18 nucleotide haplotypes (NHs). (**b**) The distribution of nucleotide polymorphism. (**c**) Diagrammatic drawing of *Pi04314* with and without signal peptide. (**d**) Sequence alignment of 14 *Pi04314* amino acid isoforms (AAIs)

**Fig. 3:**
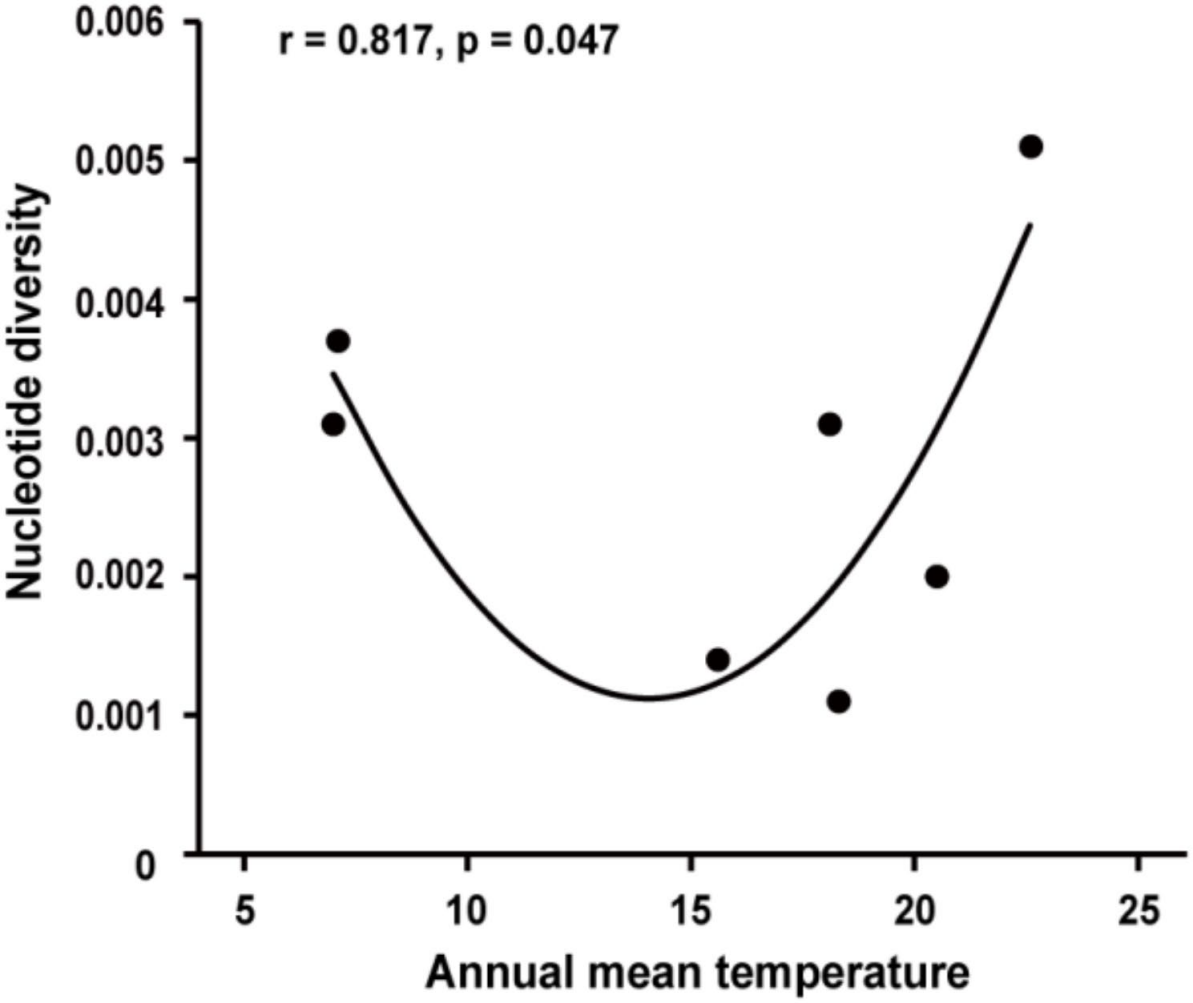
Correlation between the nucleotide diversity of *Pi04314* and the annual mean temperature of the collection sites

A total of 106 *P. infestans* isolates were sequenced for the *Pi04314* gene. The complete sequence of *Pi04314* was composed of 465 nucleotides in length, encoding an effector protein with 156 amino acids that include an N-terminal signaling peptide (SP), a conserved RXLR domain and C terminal effector domain. A total of 18 segregating sites were found in 106 full nucleotide sequences, representing 2–9 from each of the seven populations (Table 1). These variable sites formed 18 nucleotide haplotypes (NHs), with 3–7 haplotypes in each population (Table 1; Fig. 2). Nucleotide diversity of the populations ranged from 0.0011 to 0.0051 with an overall of 0.0035 when all sequences from different populations pooled together (Table 1). The lowest nucleotide diversity was found in Chongqing and Yunnan population, while the highest nucleotide diversity was found in Guangxi population. Haplotype diversity in the seven populations ranged from 0.257 to 0.817 with an overall of 0.665 (Table 1). Fujian population displayed the lowest haplotype diversity and Heilongjiang population displayed the highest haplotype diversity. The nucleotide diversity of *Pi04314* was quadratically (*r* = 0.817, *p* = 0.047, Fig. 3) but not linearly (data not shown) correlated with the annual mean temperature of the collection sites and the quadratic model contributed 67% of the variation.

**Table 1:** Sequence number, number of segregating sites in nucleotide sequence, number of haplotypes, haplotype diversity, and nucleotide diversity of *Pi04314* in seven *Phytophthora infestans* populations

Population	AT	Sample size	S	Haplotypes	Hd	Pi
Yunnan	15.6	15	2	3	0.590	0.0014
Guangxi	22.6	15	5	5	0.752	0.0051
Fujian	20.5	15	6	3	0.257	0.0020
Chongqing	18.3	15	2	3	0.457	0.0011
Hubei	18.1	15	9	5	0.476	0.0031
Ningxia	7.0	15	9	5	0.562	0.0031
Heilongjiang	7.1	16	8	7	0.817	0.0037
Total		106	18	18	0.665	0.0035

**AT**: The annual mean temperature of the collection sites; **S**: No. of segregating sites; **Hd**: Haplotype diversity; **Pi**: Nucleotide diversity

Among the 18 haplotypes, NH_1 was the most dominant. The haplotype is present in all populations, with the frequency ranged from 25 to 86.6% in each population and a grand mean of 61.3% when 106 sequences were combined (Fig. 1). NH_3, NH_8, and NH_10 present in 1–4 populations, with the frequency of 5.7–7.5% and were the second dominant haplotypes. Other haplotypes except NH_4 and NH_5 were detected only once and thus were private in one of the populations (Fig. 1).

In most of the populations including Chongqing, Hubei, Ningxia, Yunnan, and Fujian, the dominant haplotype was NH_1, while in Guangxi and Heilongjiang the dominant haplotype was NH_8 and NH_10 respectively. Haplotype frequency varied significantly among *P. infestans* populations (χ^2^ = 26.34, *p* < 0.001).

### Haplotype network of *Pi04314*

**Fig. 4:**
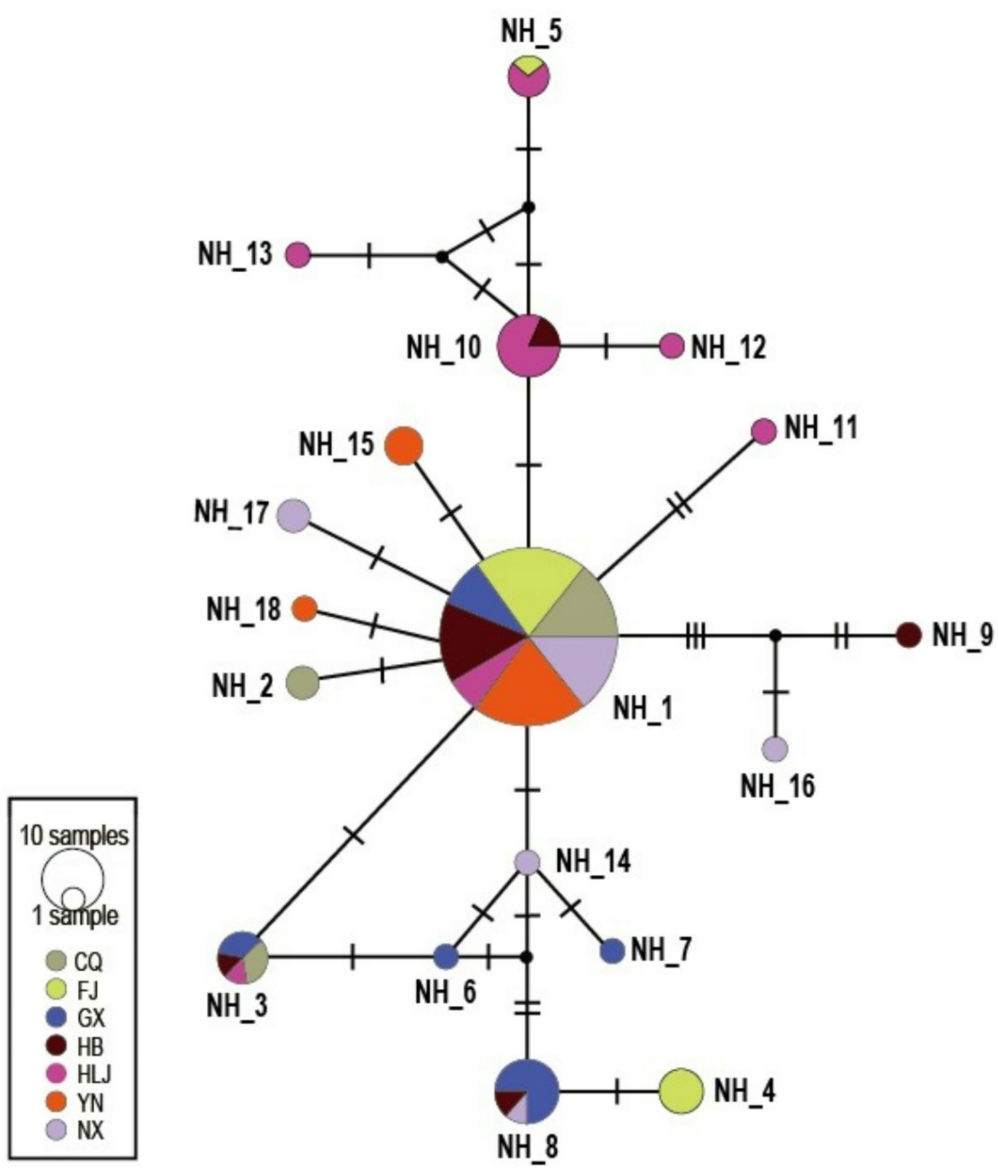
Haplotype network of *Pi04314*. Haplotype network of 18 *Pi04314* nucleotide sequences and circle sizes of the circles represent haplotype frequencies in populations

A haplotype network was generated from 18 polymorphic sites of the 18 nucleotide haplotypes. Most of the haplotypes were one to two mutation steps away from each other (Fig. 4a). In the three second dominant haplotypes, NH_3 and NH_10 had one mutation step away from the dominant haplotype NH_1 while NH_8 was genetically farther from NH_1 by four mutations. Among all populations, haplotypes from Heilongjiang and Fujian were genetically distant from the dominant haplotype. For example, NH_4, NH_5, NH_12 and NH_13 were all more than two mutation steps from NH_1. One reticulation formed by NH_1, NH_3, NH-6, and NH_14 was detected in the network. In addition, two potential reticulations were also formed by some uncaptured haplotypes (Fig. 4).

### Variation in signal peptide region of *Pi04314*

Most of the variable sites of *Pi04314* occurred in signal peptide region. Among the 18 segregating sites, 14 were present in the signal peptide region while only 4 were located in the C terminal functional regions of the effector gene (Fig. 2A). Nucleotide diversity at each nucleotide position was also calculated and it is found that almost all highly polymorphic sites were in signal peptide region (Fig. 2B, C). When translated into amino acids, the 18 nucleotide haplotypes encoded 14 amino acid isoforms (AAIs) (Fig. 2D). NH_3 and NH_15, with a synonymous mutation at nucleotide 9 and 258, respectively, were translated together with NH_1 into the same isoform AAI_1. NH_6 and NH_14, with a synonymous mutation in nucleotide 9 and 40 respectively, encoded for the same isoform AAI_5, which had a nonsynonymous mutation at amino acid 40 when compared with NH_1. Similarly, NH_12, together with NH_10, had a synonyms mutation in nucleotide 42 and encoded the same haplotype (AAI_9). This isoform has a non-synonymous mutation at 52 when aligned with NH_1. Among the 14 amino acid isoforms, 10 were generated by non-synonymous mutation in the signal peptide region.

### Functional confirmation of signal peptide mutations

**Fig. 5:**
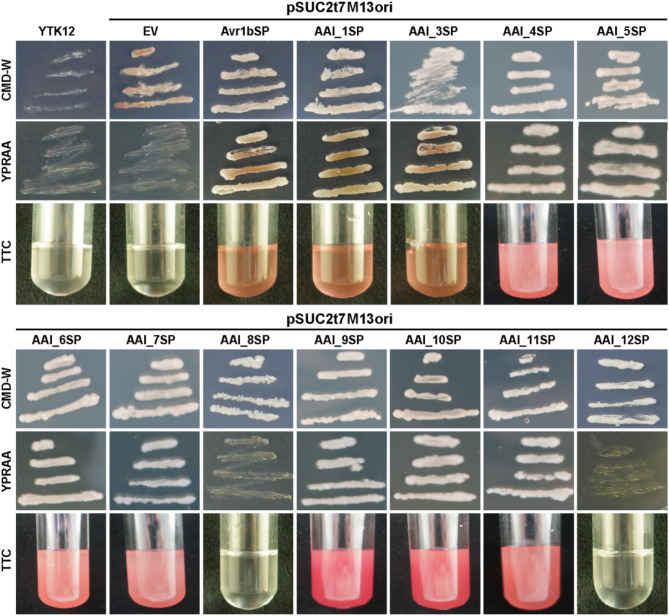
The secretion function of Pi04314 signal peptide. Yeast YTK12 strains carrying the empty vector, the signal peptide of NH_1 and Avr1b grew on the CMD-W and YPRAA plates, respectively. The enzymatic activity of invertase was detected by the reduction of 2, 3, 5-triphenyl tetrazolium chloride (TTC) to insoluble red-colored 1, 3, 5-triphenyl formazan (TPF). The negative controls are the YTK12 strain and empty vector (EV), and the positive control is the YTK12 strain carrying the signal peptide of Avr1b

SignaIP 5.0 predicts that AAI_8 loses its signal peptide function due to mutations in amino acids 6, 9, 11, 12, and 18, and AAI_12 loses tis signal peptide function due to mutations in amino acids 6, 9, 12, and 13. (Fig. 2C, D). These results were confirmed experimentally. When, the first 66 nucleotides in the 14 amino acid isoforms were cloned and tested by the yeast signal trap system and enzyme invertase, the secretory function of the signal peptides in AAI_8 and AAI_12 was lost whereas the secretory function of signal peptides in other 12 isoforms was normal despite the presence of nonsynonymous mutations in the latter (Fig. 5).

### Population differentiation in *Pi04314* and SSR marker loci

Pairwise population differentiation in *Pi04314* nucleotide sequences ranged from 0.000 to 0.370 with an overall of 0.186 when all sequences from the seven populations were pooled together (Table 2). While pairwise population differentiation in neutral SSR markers ranged from 0.041 to 0.099 with an overall of 0.074 (Table 2), which was significantly less than 0.186, the overall population differentiation in *Pi04314*nucleotides (*p* = 0.001).

**Table 2:** Pairwise population differentiation in *Pi04314* gene (below diagonal) and neutral SSR marker (above diagonal)

	Chongqing	Fujian	Guangxi	Hubei	Heilongjiang	Ningxia	Yunnan
**Chongqing**	-	0.055	0.079	0.073	0.063	0.071	0.072
**Fujian**	0.027	-	0.041	0.082	0.079	0.099	0.075
**Guangxi**	0.334	0.252	-	0.099	0.043	0.069	0.082
**Hubei**	0.002	0.000	0.214	-	0.067	0.061	0.061
**Heilongjiang**	0.263	0.168	0.342	0.135	-	0.094	0.096
**Ningxia**	0.029	0.000	0.212	0.000	0.202	-	0.065
**Yunnan**	0.089	0.052	0.370	0.038	0.277	0.048	-

### Detecting natural selection in the *Pi04314***gene**

Significantly negative Tajima’s D (-1.578, *p* = 0.040) and Fu’s FS (-10.485, *p* = 0.000) were detected in the *Pi04314* nucleotide sequences by Arlequin 3.5, suggesting that the *Pi04314* gene was undergoing purifying selection. The signal of purifying selection was also detected in 3rd codon by ω analysis using M8 model in PAML, and SLAC and REL models in HyPhy (Table 3).

**Table 3:** Putative codons in *Pi04314* sequences detected under purify selection using the PAML package and two approaches implemented in the hyphy software

Codon	PAML			HyPhy				
	Model M8			SLAC			REL	
	dN-dS	PPb		dN-dS	PPb		dN-dS	PPb
3	-20.75	0.959*		-31.8	0.95**		-21.967	0.999**

## Discussion

A total of 18 nucleotide haplotypes and 14 amino acid isoforms (AAIs) were observed from the 106 *Pi04314* sequences. All sequence variations were generated by single nucleotide substitution. While some reticulation patterns involving NH_1, N_3, NH_6 and NH_14 were found in the network, no signals of intragenic recombination were detected in the effector by further sequence analysis, suggesting the reticulation structure might be caused by other mechanisms such as convergent mutations. This pattern of low genetic variation and mutation system are similar to those detected in *Pi02860* but very different to those detected in *Avr1*,* Avr2*,* Avr3a* and *Avr4* of *P. infestans* although most of these gene sequences were generated in our lab using the same pathogen materials and the same sequencing technology [41, 49, 74–76]. For example, 65 and 51 nucleotide haplotypes were detected in the 111 and 117 sequences of *Avr2* and Avr3a, respectively. High genetic variation in *Avr1*,* Avr2*,* Avr3a* and *Avr4* can be attributed to their diverse mutation mechanisms including base substitution, altered start codon, early-termination, insertion or deletion, and intragenic recombination that has been documented in these pathogen materials. Post-translation process in addition to mutations also contributes to the higher variation of these effector genes [75].

Members of a gene family can differ significantly in sequence polymorphism, evolution and function. Like the classification of house-keeping gene or non-house-keeping gene, effector genes can also be divided into essential effectors and nonessential effectors. Essential effectors are highly conserved among and within species, are highly expressed during infection, and are indispensable for infection activity. Our analysis reveals that *Pi04314* demonstrates low genetic variation and singe mode of mutational mechanism. We believe that low genetic variation and shortage of mutation mechanism in *Pi04314* are due to lack of co-evolutionary history with its corresponding R gene in host plants. Effectors that are recognized by plants trigger host immune responses [15]. As a result, they reduce pathogen fitness and are consequently selected against during pathogen evolution. However, molecular and functional analyses indicate that many effector proteins are important factors of pathogen infection. They enhance plant susceptibility by manipulating plant cellular processes and suppressing plant defense systems [30]. Due to this counter adaptation and trade-off between the advantages and disadvantages that mutations may cause to pathogen populations, effector genes are expected to evolve differently depending on their co-evolutionary history with corresponding resistance genes in host plants, leading to a unique model of “two-speed evolution [77, 78]. When challenged by corresponding resistance genes, effector genes, i.e., *Avr1*,* Avr2*,* Avr3* and *Avr4* of *P. infestans*, could evolve at a faster rate, thereby effectively and quickly escaping host recognition systems, and increasing the invasive, survival and reproductive chances of the pathogen. Physical location in gene-sparse, transposon-rich, fast evolving regions of pathogen genome enables effectors to rapidly generate mutations through various mechanisms, such as base substitution, deletion, insertion, translocation and duplication. For example, functional redundancy encoded by multiple copies of effector genes relaxes selective pressure on one or more of the gene copies which in turn, allows more frequent and abrupt mutations to occur without severe impact on pathogen fitness. Interactions among these mutation events result in higher genetic variation in the effector genes compared to the rest of pathogen genome including other effector genes and enhances response to selection driven by the deployment of host resistance [49, 74–76]. Consequently, virulent types of effector genes can emerge quickly from avirulent types in the plant pathogen populations, leading to the suppression of effector-triggered immune responses of plants and breakdown of resistant cultivars.

However, the evolution of effector genes is constrained by the need to retain features which are critical to the biological and ecological adaptation of pathogens as shown by the severe fitness penalty of pathogens associated with unnecessary virulent effectors. For example, the maximum lesion density of *Venturia inaequalis* pathotypes with a virulent effector was 20% lower and the latent period was 7% longer compared with pathotypes without the virulent effector on apples lacking the corresponding resistance genes [79]. Similarly, when inoculated onto hosts lacking the corresponding resistance gene, ascospores from *Leposphaeria maculans* pathotypes with avirulent effectors produced more diseases than ascospores from pathotypes carrying virulent effectors [80, 81]. Furthermore, the fitness penalty of virulent mutations in effector genes can be additive or multiplicative. This unique feature also makes effector genes the best choice to study antagonistic coevolution in nature. Therefore, when effector genes, such as *Pi04314* of the study, lack coevolutionary interactions with the corresponding resistance genes in plants, natural selection tends to eliminate mutations, resulting in a signature of lower genetic variation in the populations. Indeed, when we analyzed the genomes of 100 *P. infestans* worldwide, we found that > 90% of effectors have undergone negative selection (unpublished data). The hypothesis of negative selection responsible for the lower genetic variation in *Pi04314* was also supported by the comparative analysis of synonymous and non-synonymous mutation in the gene sequences (Table 2).

Strong population differentiation was found among the effector sequences from different locations, with an overall F_ST_ of 0.186. Further analysis reveals that the spatial structure of *Pi04314* was generated by natural selection rather than genetic drift, consistent with the expectation discussed above. However, if host immunity is the only determinative force in the evolution of effector genes, when the corresponding host resistance genes are absent in field practice as the case in the current study, natural selection aiming at maximizing pathogen fitness will homogenize genetic structure of targeted gene among populations, leading to lower genetic differentiation in the *Pi04314* than neutral SSR marker loci, which has been documented in the evolution of life history traits [82]. Interestingly, when we performed a comparative population analysis of the pathogen, we found that the average genetic differentiation of *Pi04314* was significantly higher than that of SSR marker loci, suggesting that other factors besides host resistance are also involved in the evolution and local adaptation of the effector gene. It has been previously reported that ecological factors such as climatic condition also play a role in the evolution of effector genes [49, 74, 76]. Even though it is relatively weak, the quadratic rather than linear association between the nucleotide diversity of *Pi04314* and the annual mean temperature of the collection sites (Fig. 3) suggests a scenario that temperature could have a bi-directional effect on the evolution of the effector gene, consistent with previous results. More experiments, including a large number of effectors, need to be empirically tested in the future to confirm the effect of temperature on effectors.

Signal peptides are mainly responsible for directing proteins to the secretion system and ensuring that the proteins enter into target cells at the right time [83, 84]. However, recent studies indicate that signal peptides have other biological functions affecting species fitness. Although signal peptides are presumably rapidly degraded after entering a target cell, some still have functions on their own [85, 86]. In HIV, the signal peptide of the prolactin precursor (pre-Prl) is processed and accumulated in the membrane after cleavage and then released from the membrane into the cytosol. In the cytosol, the SPPrl fragments are associated with calmodulin in a Ca^2+^-dependent manner in the feedback regulation of prolactin secretion [87]. In effector genes, mutations were mainly found in functional domains, especially in C-terminals [88]. Here, we found that mutations in *Pi04314* mainly occurred in the si*g*nal peptide and two of the mutations are predicted to eliminate the pathogenicity of the pathogen by preventing effector delivery into the host cells (Fig. 5). The similar phenomena have been also detected in some effectors of other pathogens such as *BLN06* and *BSW04m* of *Bremia lactucae* [89]. However, due to the diploid nature of *P. infestans*, the lethality of the signal peptide mutations may be hidden in heterozygosity, ensuring their survival in the population.

Our results support the hypothesis that effector signal peptides may also contribute to pathogen ecological adaptation by quantitatively altering host recognition or by performing other biological functions. This hypothesis, together with the phenotypes of heterozygous signal peptide mutation in pathogen populations, can be tested molecularly in the future by generating a series of different signal peptides, synthesizing them into an effector domain and comparing their fitness with regard to pathogenicity and other functional properties of a pathogen.

In conclusion, effectors deploy a wide arrange of mutation mechanisms including base substitution, deletion, pseudogenization transposition, early-termination and transcriptional silencing [7, 49, 74, 76, 90, 91] although main mechanisms may vary among pathogens or even among effector genes within the same pathogen species. In addition, intragenic recombination and climatic factors such as temperature may also contribute to the generation and maintenance of genetic variation of effectors [76], therefore the evolution of pathogens. Interaction among these mutation and ecological events results in higher genetic variation in effector genes compared to the rest of pathogen genome and enhances response to selection driven by the change of host defense systems and ecosystem. Consequently, virulent types of effector genes can emerge quickly from avirulent types in the plant pathogen populations, rendering the effectiveness of disease-resistant cultivars and other disease management strategies.

## Conclusions

Our results reveal that a total of 18 non-synonymous mutations in *Pi04314* from the potato pathogen *P. infestans*, 15 of which were present in the signal peptide region. Two of the 14 amino acid isoforms completely abolished the secretion functions of signal peptides. The effector has undergone purifying selection, supported by the comparative analyses between its population differentiation with that of SSR marker loci as well as by negative Tajima’s D and Fu’s FS. Furthermore, we found that the nucleotide diversity of *Pi04314* is significantly correlated with the annual mean temperature of the collection sites. These results suggest that the evolution of effectors gene could be influenced by local air temperature.

## Data Availability

Data is provided within the manuscript or supplementary information files.
